# Supplementary material for: Hot spot prediction in protein-protein interactions by an ensemble system
Source: BMC Syst Biol. 2018 Dec 31;12(Suppl 9):132. doi: 10.1186/s12918-018-0665-8 (PMC6311905; doi:10.1186/s12918-018-0665-8)
Supplement: Supplementary file 2 — All relevant MATLAB code. A simple Matlab implement of our predictor. (ZIP 19,742 kb) [file 12918_2018_665_MOESM2_ESM.zip › Additional file 2/Read ASA/ASA website/dbMPIKT/3BE1H.html]

 NetSurfP - Protein Surface Accessibility and Secondary Structure Predictions

  

|  |  |  |
| --- | --- | --- |
|  |  | NetSurfP - Protein Surface Accessibility and Secondary Structure Predictions  Technical University of Denmark |

  


---

```
# For publication of results, please cite:
# A generic method for assignment of reliability scores applied to solvent accessibility predictions.
# Bent Petersen, Thomas Nordahl Petersen, Pernille Andersen, Morten Nielsen and Claus Lundegaard
# BMC Structural Biology 2009, 9:51 doi:10.1186/1472-6807-9-51
#
# Column 1: Class assignment - B for buried or E for Exposed - Threshold: 25% exposure, but not based on RSA
# Column 2: Amino acid
# Column 3: Sequence name
# Column 4: Amino acid number
# Column 5: Relative Surface Accessibility - RSA
# Column 6: Absolute Surface Accessibility
# Column 7: Z-fit score for RSA prediction
# Column 8: Probability for Alpha-Helix
# Column 9: Probability for Beta-strand
# Column 10: Probability for Coil
E E  3BE1H                  1    0.798 139.411   1.316   0.003   0.003   0.994
B I  3BE1H                  2    0.264  48.914  -0.794   0.019   0.141   0.840
B S  3BE1H                  3    0.173  20.299  -0.724   0.021   0.279   0.699
E E  3BE1H                  4    0.407  71.138   0.631   0.021   0.451   0.528
B V  3BE1H                  5    0.128  19.704   0.534   0.021   0.756   0.223
E Q  3BE1H                  6    0.358  63.903   1.057   0.018   0.846   0.136
B L  3BE1H                  7    0.110  20.086   0.782   0.018   0.846   0.136
E V  3BE1H                  8    0.354  54.364   0.626   0.023   0.655   0.322
B E  3BE1H                  9    0.244  42.539   0.742   0.021   0.451   0.528
E S  3BE1H                 10    0.417  48.837  -0.476   0.019   0.141   0.840
E G  3BE1H                 11    0.507  39.869  -1.844   0.018   0.047   0.935
E G  3BE1H                 12    0.535  42.065  -2.289   0.018   0.047   0.935
E G  3BE1H                 13    0.527  41.459  -1.757   0.019   0.141   0.840
E L  3BE1H                 14    0.406  74.247  -0.877   0.022   0.359   0.619
E V  3BE1H                 15    0.298  45.864   0.631   0.021   0.451   0.528
E Q  3BE1H                 16    0.425  75.834   1.158   0.020   0.205   0.775
E P  3BE1H                 17    0.466  66.140  -0.578   0.018   0.047   0.935
E G  3BE1H                 18    0.611  48.062  -2.079   0.005   0.045   0.951
E G  3BE1H                 19    0.418  32.928  -0.690   0.004   0.138   0.858
E S  3BE1H                 20    0.430  50.373  -0.475   0.004   0.420   0.576
B L  3BE1H                 21    0.151  27.740   0.838   0.002   0.816   0.182
E R  3BE1H                 22    0.411  94.096   0.800   0.001   0.900   0.099
B L  3BE1H                 23    0.074  13.623   0.328   0.001   0.900   0.099
B S  3BE1H                 24    0.169  19.854   0.651   0.001   0.900   0.099
B C  3BE1H                 25    0.038   5.279  -0.111   0.001   0.900   0.099
B A  3BE1H                 26    0.289  31.826   0.661   0.001   0.900   0.099
B A  3BE1H                 27    0.103  11.329  -0.256   0.002   0.816   0.182
B S  3BE1H                 28    0.173  20.252  -0.517   0.004   0.514   0.481
B G  3BE1H                 29    0.231  18.195  -1.924   0.004   0.197   0.799
B F  3BE1H                 30    0.244  48.891  -0.903   0.005   0.336   0.660
B N  3BE1H                 31    0.170  24.961  -1.362   0.004   0.514   0.481
B I  3BE1H                 32    0.116  21.552  -0.553   0.021   0.451   0.528
B K  3BE1H                 33    0.209  43.012  -1.060   0.021   0.279   0.699
B D  3BE1H                 34    0.298  42.899  -1.299   0.018   0.088   0.893
B T  3BE1H                 35    0.121  16.769  -0.268   0.019   0.141   0.840
B Y  3BE1H                 36    0.094  19.981   0.083   0.022   0.359   0.619
B I  3BE1H                 37    0.030   5.550   0.082   0.023   0.655   0.322
B H  3BE1H                 38    0.050   9.150  -0.143   0.021   0.756   0.223
B W  3BE1H                 39    0.035   8.345   0.501   0.018   0.846   0.136
B V  3BE1H                 40    0.037   5.656   0.303   0.018   0.846   0.136
B R  3BE1H                 41    0.118  27.022   0.356   0.021   0.756   0.223
B Q  3BE1H                 42    0.091  16.270   0.363   0.021   0.451   0.528
B A  3BE1H                 43    0.205  22.635   0.821   0.005   0.045   0.951
E P  3BE1H                 44    0.378  53.667   0.781   0.005   0.015   0.979
E G  3BE1H                 45    0.597  46.945  -1.825   0.018   0.019   0.964
E K  3BE1H                 46    0.411  84.563   0.642   0.018   0.019   0.964
E G  3BE1H                 47    0.457  35.997  -0.428   0.018   0.088   0.893
B L  3BE1H                 48    0.159  29.058   0.895   0.005   0.336   0.660
B E  3BE1H                 49    0.110  19.234  -0.199   0.003   0.718   0.279
B W  3BE1H                 50    0.048  11.568   0.117   0.001   0.900   0.099
B V  3BE1H                 51    0.025   3.904   0.828   0.001   0.959   0.040
B A  3BE1H                 52    0.017   1.873  -0.042   0.001   0.959   0.040
B R  3BE1H                 53    0.081  18.435  -0.662   0.000   0.983   0.017
B I  3BE1H                 54    0.023   4.347   0.770   0.001   0.959   0.040
B Y  3BE1H                 55    0.154  32.867   0.549   0.002   0.816   0.182
B P  3BE1H                 56    0.272  38.654  -0.910   0.005   0.336   0.660
E T  3BE1H                 57    0.540  74.912  -1.655   0.005   0.045   0.951
E N  3BE1H                 58    0.605  88.631  -2.750   0.003   0.003   0.994
E G  3BE1H                 59    0.524  41.247  -2.345   0.018   0.019   0.964
E Y  3BE1H                 60    0.489 104.563  -0.589   0.018   0.088   0.893
E T  3BE1H                 61    0.309  42.914  -0.037   0.020   0.205   0.775
E R  3BE1H                 62    0.428  97.897   0.041   0.021   0.279   0.699
B Y  3BE1H                 63    0.203  43.445   0.333   0.020   0.205   0.775
B A  3BE1H                 64    0.215  23.737  -0.146   0.052   0.084   0.864
E D  3BE1H                 65    0.627  90.293  -0.647   0.053   0.043   0.903
E S  3BE1H                 66    0.380  44.524  -0.089   0.018   0.047   0.935
B V  3BE1H                 67    0.077  11.804  -0.532   0.019   0.141   0.840
E K  3BE1H                 68    0.372  76.438   0.796   0.020   0.205   0.775
B G  3BE1H                 69    0.207  16.267  -1.472   0.005   0.336   0.660
B R  3BE1H                 70    0.204  46.693   0.778   0.003   0.718   0.279
B F  3BE1H                 71    0.051  10.155  -0.287   0.001   0.900   0.099
B T  3BE1H                 72    0.110  15.271   0.629   0.001   0.959   0.040
B I  3BE1H                 73    0.042   7.696   0.595   0.001   0.959   0.040
B S  3BE1H                 74    0.090  10.595   0.616   0.002   0.816   0.182
B A  3BE1H                 75    0.247  27.219   0.692   0.004   0.616   0.381
B D  3BE1H                 76    0.205  29.555  -0.465   0.004   0.514   0.481
E T  3BE1H                 77    0.424  58.739  -0.212   0.018   0.088   0.893
B S  3BE1H                 78    0.272  31.867  -1.873   0.018   0.047   0.935
E K  3BE1H                 79    0.525 108.054   0.685   0.018   0.088   0.893
B N  3BE1H                 80    0.249  36.424  -0.512   0.022   0.359   0.619
B T  3BE1H                 81    0.211  29.224   0.991   0.018   0.846   0.136
B A  3BE1H                 82    0.071   7.813  -0.499   0.011   0.918   0.071
B Y  3BE1H                 83    0.163  34.790   0.543   0.006   0.962   0.032
B L  3BE1H                 84    0.047   8.642   0.544   0.011   0.918   0.071
B Q  3BE1H                 85    0.159  28.362  -0.450   0.011   0.918   0.071
B M  3BE1H                 86    0.065  13.006  -0.759   0.023   0.655   0.322
E N  3BE1H                 87    0.374  54.827  -0.046   0.022   0.359   0.619
E S  3BE1H                 88    0.392  45.919  -0.646   0.020   0.205   0.775
B L  3BE1H                 89    0.114  20.892   0.054   0.018   0.088   0.893
E R  3BE1H                 90    0.485 111.065   0.995   0.018   0.088   0.893
E A  3BE1H                 91    0.376  41.402  -0.561   0.052   0.084   0.864
E E  3BE1H                 92    0.437  76.344  -0.702   0.053   0.043   0.903
B D  3BE1H                 93    0.217  31.270  -0.785   0.052   0.084   0.864
B T  3BE1H                 94    0.101  13.995   0.175   0.066   0.296   0.638
B A  3BE1H                 95    0.035   3.879  -0.338   0.085   0.775   0.140
B V  3BE1H                 96    0.060   9.283   0.975   0.072   0.855   0.073
B Y  3BE1H                 97    0.038   8.014   0.604   0.072   0.855   0.073
B Y  3BE1H                 98    0.048  10.364   0.778   0.085   0.775   0.140
B C  3BE1H                 99    0.016   2.274   1.438   0.023   0.655   0.322
B S  3BE1H                100    0.030   3.516  -0.102   0.022   0.552   0.426
B R  3BE1H                101    0.126  28.900  -0.108   0.021   0.451   0.528
B W  3BE1H                102    0.222  53.487  -0.482   0.066   0.296   0.638
E G  3BE1H                103    0.323  25.444  -1.286   0.019   0.141   0.840
E G  3BE1H                104    0.387  30.457  -2.333   0.018   0.047   0.935
E D  3BE1H                105    0.370  53.375  -1.529   0.018   0.047   0.935
E G  3BE1H                106    0.373  29.379  -2.071   0.018   0.047   0.935
E F  3BE1H                107    0.309  61.956  -1.637   0.018   0.088   0.893
B Y  3BE1H                108    0.240  51.309  -0.574   0.018   0.088   0.893
B A  3BE1H                109    0.119  13.147  -1.123   0.019   0.141   0.840
B M  3BE1H                110    0.064  12.846   0.032   0.020   0.205   0.775
B D  3BE1H                111    0.095  13.632  -0.652   0.020   0.205   0.775
B Y  3BE1H                112    0.074  15.750  -0.379   0.020   0.205   0.775
B W  3BE1H                113    0.050  12.097  -0.969   0.019   0.141   0.840
B G  3BE1H                114    0.048   3.778  -1.819   0.019   0.141   0.840
B Q  3BE1H                115    0.156  27.808  -0.060   0.004   0.085   0.910
B G  3BE1H                116    0.120   9.436  -1.743   0.005   0.336   0.660
B T  3BE1H                117    0.072   9.945   0.035   0.004   0.616   0.381
E L  3BE1H                118    0.219  40.026   0.202   0.001   0.900   0.099
B V  3BE1H                119    0.034   5.303   0.267   0.001   0.900   0.099
B T  3BE1H                120    0.085  11.803   0.396   0.001   0.900   0.099
B V  3BE1H                121    0.066  10.129   0.213   0.002   0.816   0.182
B S  3BE1H                122    0.127  14.908  -0.431   0.004   0.616   0.381
B S  3BE1H                123    0.239  28.011  -1.075   0.004   0.197   0.799
E A  3BE1H                124    0.467  51.452  -1.795   0.018   0.047   0.935
E S  3BE1H                125    0.447  52.447  -0.436   0.018   0.047   0.935
E T  3BE1H                126    0.458  63.580  -1.473   0.018   0.047   0.935
E K  3BE1H                127    0.633 130.290  -1.397   0.018   0.047   0.935
E G  3BE1H                128    0.533  41.939  -1.938   0.005   0.015   0.979
E P  3BE1H                129    0.415  58.945  -1.887   0.005   0.015   0.979
E S  3BE1H                130    0.546  63.979  -1.408   0.004   0.138   0.858
E V  3BE1H                131    0.316  48.538  -1.826   0.005   0.262   0.733
B F  3BE1H                132    0.187  37.451   0.155   0.005   0.262   0.733
E P  3BE1H                133    0.344  48.771  -1.026   0.005   0.262   0.733
B L  3BE1H                134    0.198  36.162  -1.570   0.004   0.197   0.799
E A  3BE1H                135    0.384  42.317  -1.382   0.004   0.138   0.858
B P  3BE1H                136    0.277  39.235  -1.924   0.004   0.085   0.910
B S  3BE1H                137    0.213  24.928  -2.546   0.005   0.045   0.951
E S  3BE1H                138    0.474  55.588  -2.658   0.005   0.045   0.951
E K  3BE1H                139    0.691 142.159  -2.160   0.005   0.015   0.979
E S  3BE1H                140    0.448  52.470  -2.300   0.005   0.015   0.979
E T  3BE1H                141    0.458  63.538  -1.399   0.003   0.003   0.994
E S  3BE1H                142    0.499  58.483  -2.000   0.003   0.003   0.994
E G  3BE1H                143    0.662  52.107  -1.585   0.016   0.005   0.979
E G  3BE1H                144    0.443  34.872  -1.520   0.018   0.047   0.935
E T  3BE1H                145    0.421  58.337   0.293   0.005   0.336   0.660
B A  3BE1H                146    0.074   8.111   0.279   0.002   0.816   0.182
B A  3BE1H                147    0.235  25.941   0.699   0.001   0.900   0.099
B L  3BE1H                148    0.052   9.485  -0.358   0.001   0.900   0.099
B G  3BE1H                149    0.055   4.352   0.073   0.001   0.900   0.099
B C  3BE1H                150    0.035   4.886  -0.531   0.002   0.816   0.182
B L  3BE1H                151    0.169  30.981   0.200   0.001   0.900   0.099
B V  3BE1H                152    0.072  11.113  -0.855   0.002   0.816   0.182
B K  3BE1H                153    0.269  55.251   0.048   0.004   0.616   0.381
E D  3BE1H                154    0.364  52.452  -1.842   0.004   0.420   0.576
B Y  3BE1H                155    0.231  49.301  -0.701   0.004   0.420   0.576
E F  3BE1H                156    0.326  65.388  -0.498   0.005   0.262   0.733
B P  3BE1H                157    0.245  34.780  -0.835   0.004   0.197   0.799
E E  3BE1H                158    0.559  97.657  -0.471   0.004   0.138   0.858
E P  3BE1H                159    0.362  51.325  -1.476   0.004   0.197   0.799
B V  3BE1H                160    0.200  30.725  -0.030   0.003   0.718   0.279
E T  3BE1H                161    0.308  42.789  -0.130   0.001   0.900   0.099
B V  3BE1H                162    0.053   8.131   0.457   0.011   0.918   0.071
B S  3BE1H                163    0.218  25.538   0.264   0.011   0.918   0.071
B W  3BE1H                164    0.081  19.384  -0.245   0.011   0.918   0.071
E N  3BE1H                165    0.354  51.884   0.436   0.018   0.846   0.136
E S  3BE1H                166    0.498  58.401  -0.462   0.004   0.420   0.576
E G  3BE1H                167    0.584  45.992  -0.973   0.018   0.088   0.893
E A  3BE1H                168    0.613  67.553  -0.579   0.005   0.015   0.979
E L  3BE1H                169    0.492  90.140  -0.417   0.018   0.047   0.935
E T  3BE1H                170    0.565  78.365  -2.477   0.005   0.045   0.951
E S  3BE1H                171    0.553  64.835  -0.720   0.005   0.045   0.951
E G  3BE1H                172    0.497  39.106  -0.730   0.005   0.045   0.951
B V  3BE1H                173    0.181  27.758  -1.080   0.005   0.262   0.733
E H  3BE1H                174    0.452  82.164   0.130   0.003   0.718   0.279
E T  3BE1H                175    0.360  49.960  -0.427   0.003   0.718   0.279
B F  3BE1H                176    0.206  41.344   0.939   0.022   0.552   0.426
B P  3BE1H                177    0.170  24.123  -0.613   0.021   0.279   0.699
B A  3BE1H                178    0.278  30.625  -1.260   0.022   0.359   0.619
B V  3BE1H                179    0.281  43.220  -0.747   0.023   0.655   0.322
B L  3BE1H                180    0.258  47.313  -0.932   0.004   0.616   0.381
E Q  3BE1H                181    0.425  75.941  -0.923   0.004   0.420   0.576
E S  3BE1H                182    0.582  68.152  -2.620   0.004   0.138   0.858
E S  3BE1H                183    0.593  69.488  -2.097   0.005   0.045   0.951
E G  3BE1H                184    0.490  38.571  -2.140   0.004   0.085   0.910
E L  3BE1H                185    0.390  71.391  -0.589   0.005   0.336   0.660
B Y  3BE1H                186    0.244  52.186  -1.108   0.004   0.616   0.381
B S  3BE1H                187    0.159  18.635  -1.367   0.003   0.718   0.279
B L  3BE1H                188    0.178  32.610  -0.905   0.002   0.816   0.182
B S  3BE1H                189    0.132  15.494  -0.364   0.001   0.900   0.099
B S  3BE1H                190    0.058   6.821  -0.883   0.001   0.900   0.099
B V  3BE1H                191    0.119  18.352   0.163   0.001   0.900   0.099
B V  3BE1H                192    0.127  19.597  -0.315   0.001   0.900   0.099
B T  3BE1H                193    0.181  25.146   0.409   0.002   0.816   0.182
B V  3BE1H                194    0.142  21.795   0.826   0.003   0.718   0.279
E P  3BE1H                195    0.328  46.515  -0.218   0.021   0.451   0.528
E S  3BE1H                196    0.386  45.298  -0.443   0.020   0.205   0.775
E S  3BE1H                197    0.588  68.949  -0.278   0.021   0.279   0.699
E S  3BE1H                198    0.687  80.493   0.327   0.020   0.205   0.775
B L  3BE1H                199    0.278  50.902  -0.625   0.018   0.088   0.893
E G  3BE1H                200    0.685  53.886   0.266   0.005   0.015   0.979
E T  3BE1H                201    0.631  87.492   0.357   0.005   0.015   0.979
E Q  3BE1H                202    0.547  97.676   0.907   0.005   0.262   0.733
E T  3BE1H                203    0.501  69.530   1.416   0.002   0.816   0.182
B Y  3BE1H                204    0.066  14.168   0.860   0.001   0.959   0.040
E I  3BE1H                205    0.309  57.091   1.914   0.001   0.959   0.040
B C  3BE1H                206    0.023   3.229   0.542   0.001   0.959   0.040
E N  3BE1H                207    0.347  50.801   1.160   0.001   0.959   0.040
B V  3BE1H                208    0.074  11.435  -0.176   0.001   0.900   0.099
E N  3BE1H                209    0.517  75.703   0.517   0.001   0.900   0.099
B H  3BE1H                210    0.197  35.871   0.460   0.004   0.616   0.381
E K  3BE1H                211    0.623 128.192   0.206   0.005   0.045   0.951
E P  3BE1H                212    0.771 109.433  -1.313   0.005   0.015   0.979
E S  3BE1H                213    0.503  58.963  -0.616   0.005   0.045   0.951
E N  3BE1H                214    0.539  78.895  -0.226   0.004   0.085   0.910
E T  3BE1H                215    0.449  62.235   0.434   0.005   0.262   0.733
E K  3BE1H                216    0.568 116.796   1.175   0.003   0.718   0.279
B V  3BE1H                217    0.107  16.446   0.109   0.002   0.816   0.182
E D  3BE1H                218    0.634  91.359  -0.108   0.001   0.900   0.099
E K  3BE1H                219    0.258  52.988   1.043   0.001   0.900   0.099
E K  3BE1H                220    0.589 121.240   0.567   0.002   0.816   0.182
B V  3BE1H                221    0.165  25.376   0.511   0.004   0.514   0.481
E E  3BE1H                222    0.478  83.454  -0.178   0.004   0.197   0.799
E P  3BE1H                223    0.549  77.917  -1.437   0.018   0.088   0.893
E K  3BE1H                224    0.599 123.297  -0.366   0.018   0.019   0.964
E S  3BE1H                225    0.363  42.497  -0.947   0.003   0.003   0.994
E C  3BE1H                226    0.394  55.374  -1.868   0.003   0.003   0.994
E D  3BE1H                227    0.582  83.866  -1.883   0.003   0.003   0.994
E K  3BE1H                228    0.603 123.934  -0.057   0.003   0.003   0.994
E T  3BE1H                229    0.535  74.135  -1.675   0.005   0.015   0.979
E H  3BE1H                230    0.800 145.484  -1.799   0.003   0.003   0.994

Explain the output.  Go back.
```

---
